# PROFESSOR PAULO ROBERTO SAVASSI ROCHA - FORMER PRESIDENT OF THE BRAZILIAN COLLEGE OF DIGESTIVE SURGERY

**DOI:** 10.1590/0102-672020230044e1762

**Published:** 2023-10-13

**Authors:** Samir RASSLAN

**Affiliations:** 1 Senior Professor, Department of Surgery. Faculty of Medicine, Universidade de São Paulo, Sao Paulo (SP), Brazil. Former President of the Brazilian College of Surgeons (1995–1997).


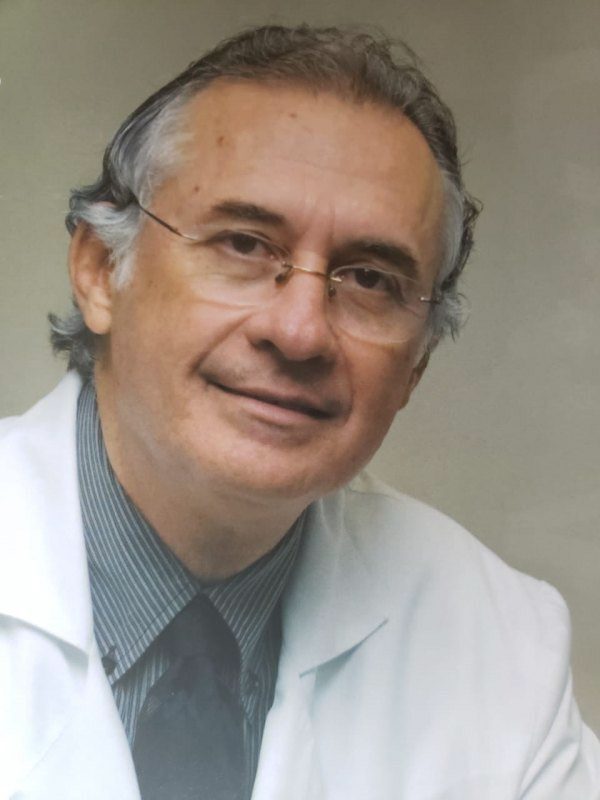


My first contact with Professor Paulo Roberto Savassi Rocha was in November 1983, therefore exactly 40 years ago, when I went to Belo Horizonte to participate in the Judging Committee for the defense of his doctoral thesis, at Universidade Federal de Minas Gerais (UFMG), at the invitation of the late Professor Alcino Lázaro da Silva^1^.

As I didn’t know the candidate, I requested Professor Alcino to ask him to send me a summary of his Curriculum Vitae, so that I could know who the Post graduate student was, author of the work I was going to analyze.

I must confess that I was impressed with his publications and two edited books. He was a surgeon who was already qualified and linked to the University.

The thesis, entitled Determination of the optimal points for resection of ischemic intestinal loops by “Dopplerometry, Thermometry and Fluoresceinoscopy: Experimental study in dogs”, analyzed a topic — intestinal ischemia and extensive resections — in which I was very interested^2^. Once again, I was impressed with the clarity and didactics in the presentation of the work, as well as with the security and propriety in the answers to the formulated questions.

During the flight back to São Paulo, in the company of Doctor Emil Burihan, Full Professor of Vascular Surgery at Escola Paulista de Medicina, now Universidade Federal de São Paulo (UNIFESP), also a member of the Judging Committee, we talked about the candidate and the thesis argument and concluded that we were witnessing the birth of a future great professor at UFMG.

Doctor Paulo Roberto Savassi Rocha is a *mineiro* in heart and soul and, like any good *mineiro*, enchanted by his land, its history, and its values. He graduated from UFMG in 1969.

After graduation, he worked for a period as an assistant to Professor Mahrdas Salvador Nankran, a renowned surgeon from Belo Horizonte, who contributed a lot to his training and growth. He then took the exam, being admitted as a resident of General Surgery at the Hospital das Clínicas of UFMG, in Belo Horizonte. After completing the residency program, he took another exam, this time joining as Teaching Instructor of the Surgery Service. He then enrolled in the Postgraduate Course (*stricto sensu*), defended his thesis and had a rapid and fulminant evolution in his academic career, reaching the position of Full Professor of Surgery in 1991.

Its trajectory is very rich. In a short period of time, he became a well-known surgeon throughout Brazil. He is a constant figure in the most important surgical events, with dozens and dozens of lectures both in the country and abroad, being invited to numerous participations in examining committees of postgraduate theses and competitions in the academic career in several institutions in the country.

For more than four decades he was a professor at the university, with intense administrative and technical-scientific activities, including:

- advisor professor of the Post Graduate Program in Sciences Applied to Surgery;
- effective member of the Board of the Post Graduate Program in Surgery;
- chairman of the Research Committee of the Department of Surgery;
- member of the Commission for the Project and Implementation of the Outpatient Surgery Sector;
- director of the division of Digestive Tract and Gastrointestinal Surgery
- supervisor of over 40 Master’s and Doctoral theses;
- actively participated in the training of a significant number of surgeons and residents in General Surgery and Digestive System;
- author of more than 30 scientific books on topics in Gastroenterology, Fundamentals of Clinical Surgery, Surgical Emergencies, Complications in Surgery of the Digestive Tract;
- Enthusiastic about the use of videosurgery since its inception in the 1990s, he was one of the first surgeons in the country to publish the experience with laparoscopic cholecystectomy^3,4,5^.

In 2002, he inaugurated with his great partner, Professor Aloísio de Paula Castro, the *Instituto Alfa de Gastroenterologia*, one of the most complete and important Services for Diseases of the Digestive System in the Country.

He received numerous honors and decorations. To name just a few of them:

- State Legislative Merit Medal, awarded by the Legislative Assembly of Minas Gerais in 2010;
- Medical Personality of the Year, granted by the Medical Association of Minas Gerais in 2011;
- Medical Excellence Award from the Academia Mineira de Medicina, in 2012;
- Diploma of Honor of Merit from the Medical Association, the Regional Council and the Union of Doctors of the State of Minas Gerais, in 2019;
- honored several times by graduating students of the Medicine Course at the Federal University of Minas Gerais.

His activities in medical associations were also intense:

- he held several boards and was president, in the 2002–2003 term, of the Brazilian College of Digestive Surgery;
- full member and founder of the Brazilian Association of Gastric Cancer;
- sector vice-president of the Brazilian College of Surgeons (1995–1997).

However, the most important moment of his academic life occurred in March 2016, when he received the title of Professor Emeritus of the School of Medicine of Universidade Federal de Minas Gerais, which crowned his path of successes and achievements.

Professor Savassi Rocha, in addition to being an excellent doctor, surgeon, and teacher, is also a writer. He found this other path in his rich existence.

In addition to his more than 30 medical books, he wrote two of poetry, and one of short stories: *Catarse* in 1993, *Desenredo* in 2017 (poetry), and *Intercadência* in 2019 (short stories), books that overflow his emotions and feelings. As it is said in the works, “poetry touches the soul and stories assess the fundamental values of human existence”.

I cannot fail to mention that his main literary reference is João Guimarães Rosa, from Minas Gerais, whom he considers the greatest of all writers, the great genius of literature. Savassi Rocha is an ardent “Rosean”^1^!

Professor Paulo Roberto Savassi Rocha is one of the most brilliant minds I have had the pleasure of meeting. I enjoyed the privilege of working together in the National Directory of the Brazilian College of Surgeons. He, always calm, thoughtful, and a peacemaker. I have followed his trajectory for 40 years, his personal life and his academic career. We were together on countless occasions. I have deep respect and admiration for him. Simple man, cultured, educated, elegant and in love with his family.

I would like to thank the Brazilian College of Digestive Surgery for the privilege and honor of being invited to write about Professor Paulo Roberto Savassi Rocha, who belongs to the great field of Medicine in Minas Gerais and who enriches the gallery of national Surgery.
